# Sézary‐Like Features in Atypical Vacuolated Cells of Adult T‐Cell Leukemia/Lymphoma

**DOI:** 10.1002/jha2.70024

**Published:** 2025-03-20

**Authors:** Radu Chiriac, Lucile Baseggio

**Affiliations:** ^1^ Hematology Laboratory Hospices Civils de Lyon, Centre Hospitalier Lyon Sud Lyon France

**Keywords:** adult T‐cell leukemia/lymphoma, cytology, HTLV‐1, CD25

1

A man in his 40s presented with a 2‐week history of fatigue, headaches, diffuse abdominal pain, and sweating, without fever. Clinical examination revealed multiple bilateral cervical and inguinal lymphadenopathies, splenomegaly, and a back rash without evidence of papules. Additionally, a whole‐body CT scan demonstrated extensive lymphadenopathy above and below the diaphragm, as well as lytic bone lesions in the pelvis.

Laboratory investigations revealed hypercalcemia (3.2 mmol/L), elevated lactate dehydrogenase (2000 U/L), anemia (78 g/L), and leukocytosis (95 × 10⁹/L) with 80% monomorphic atypical lymphoid cells, characterized by folded chromatin nuclei, weakly basophilic cytoplasm, and rare vacuoles (Figure 1A). Additionally, HTLV‐1 serology was positive.

**FIGURE 1:**
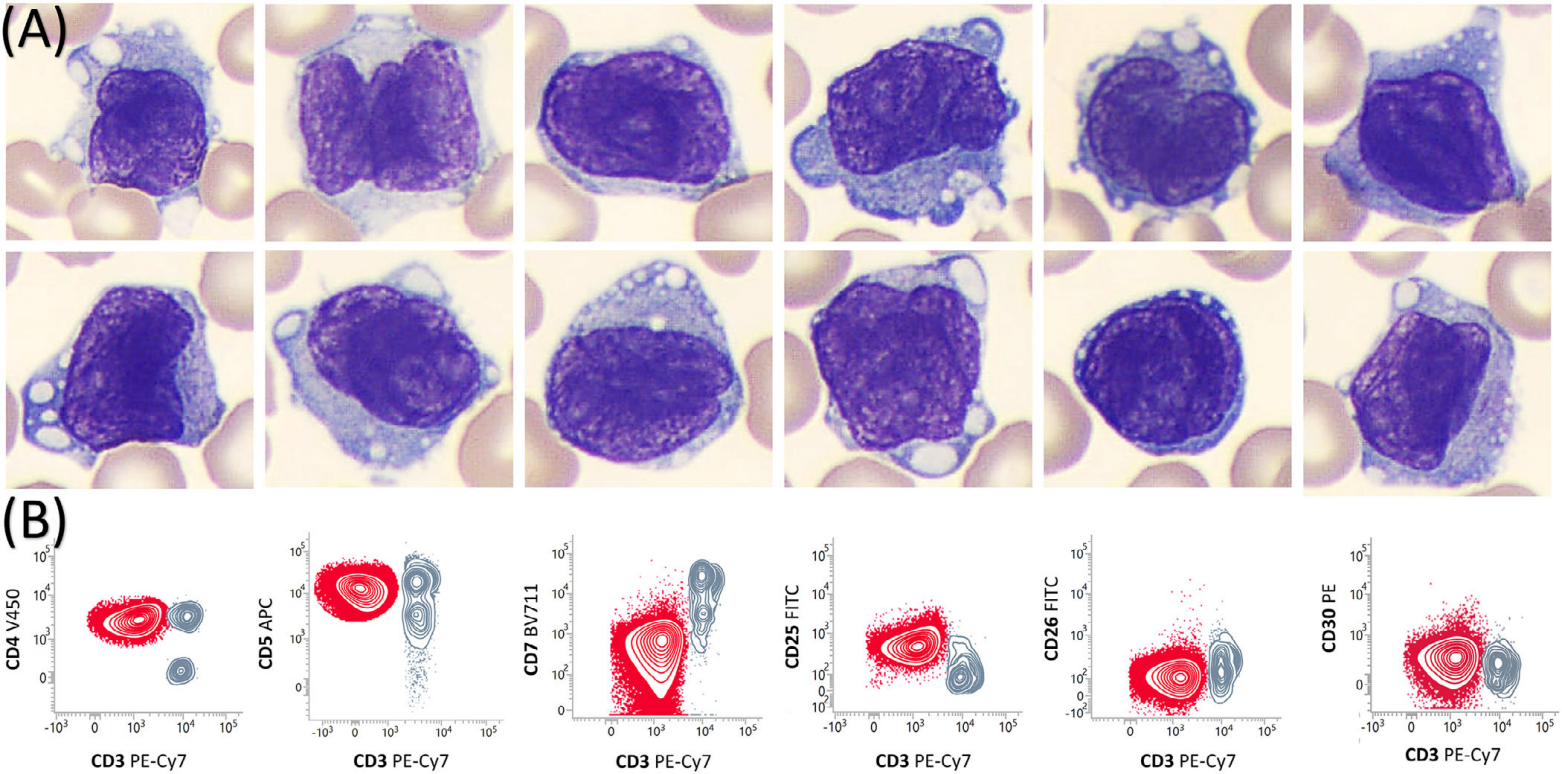
Panel A: May‐Grunwald Giemsa stain, ×100 objective, showing monomorphic atypical lymphoid cells, characterized by folded chromatin nuclei, weakly basophilic cytoplasm, and rare vacuoles. Panel B: Flow cytometry of peripheral blood (red population) identified a CD4+ T‐cell population that showed loss of surface CD3, CD5+, CD7‐, CD25+, CD26‐, and weak expression of CD30+.

Flow cytometry of peripheral blood (Figure 1B, red population) identified a CD4+ T‐cell population that had lost surface CD3, with weak CD2, weak CD30, strong CD5, and no CD7 expression. The cells expressed CD25 and were negative for CD26, KIR3DL2, CCR4, and T follicular helper markers (ICOS, CXCR5, PD1). Further, an inguinal lymph node biopsy demonstrating the same phenotypic profile strongly supported the diagnosis of adult T‐cell leukemia/lymphoma (ATLL).

Extensive lymphoma infiltration of the bone marrow was observed, with no evidence of central nervous system involvement. Conventional bone marrow karyotyping revealed no chromosomal abnormalities and targeted next‐generation sequencing of the lymph node identified mutations in the *TP53* (variant allele frequency [VAF] 32%) and *PLCG1* (VAF 36%) genes.

After the third cycle of Brentuximab vedotin in combination with cyclophosphamide, doxorubicin, etoposide, and prednisone, there was no clinical, microscopic, or radiographic evidence of disease, according to the adapted Lugano and ATLL staging criteria. In complete remission, a plan for consolidation therapy involving allogeneic stem cell transplantation is currently being pursued.

This case highlights the morphological pleomorphism observed in an acute leukemic presentation of ATLL. While medium to large‐sized lymphocytes with multilobulated nuclei, commonly referred to as “flower cells,” are a characteristic feature in the acute form of the disease, the present case presents lymphocytes with convoluted nuclei, a morphology more frequently associated with Sézary syndrome. Furthermore, the identification of documented variants, including chronic lymphocytic leukemia‐like morphology and prolymphocytic features, emphasizes the considerable morphological variability inherent to this entity [1, 2].

In addition to the detection of integrated human T‐cell leukemia virus type 1 in isolated lymphoma cells or the presence of anti‐human T‐cell leukemia virus type 1/2 antibodies in the serum, which serve as imperfect surrogates, ATLL lacks distinct clinical, morphological, immunophenotypic, or molecular characteristics. The variability in clinical presentations, the absence of specific molecular markers, and the rarity of the virus in nonendemic populations make it difficult to accurately identify certain subsets of ATLL, particularly the lymphomatous and smoldering/cutaneous forms.

## Author Contributions

Radu Chiriac wrote the manuscript and performed the cytological studies, and Lucile Baseggio conducted the flow cytometric studies. All authors contributed to the final manuscript.

## Ethics Statement

This manuscript respects the ethical policy of CHU Lyon for the treatment of human research participants.

## Consent

The authors have confirmed that a patient consent statement is not required for this submission as no patient‐identifying data were used.

## Conflicts of Interest

The authors declare no conflicts of interest.

## Permission to Reproduce Material From Other Sources

The authors declare no use of third‐party material in this study for which formal permission is required.

## Data Availability

Data sharing is not applicable to this article as no new data were created or analyzed in this study.
